# An integrated approach to the prediction of domain-domain interactions

**DOI:** 10.1186/1471-2105-7-269

**Published:** 2006-05-25

**Authors:** Hyunju Lee, Minghua Deng, Fengzhu Sun, Ting Chen

**Affiliations:** 1 Department of Computer Science, University of Southern California, Los Angeles, CA 90089, USA; 2 School of Mathematical Sciences and Center for Theoretical Biology, Peking University, Beijing 100871, P.R. China; 3 Molecular and Computational Biology Program, Department of Biological Sciences, University of Southern California, 1050 Childs Way, Los Angeles, CA 90089-2910, USA

## Abstract

**Background:**

The development of high-throughput technologies has produced several large scale protein interaction data sets for multiple species, and significant efforts have been made to analyze the data sets in order to understand protein activities. Considering that the basic units of protein interactions are domain interactions, it is crucial to understand protein interactions at the level of the domains. The availability of many diverse biological data sets provides an opportunity to discover the underlying domain interactions within protein interactions through an integration of these biological data sets.

**Results:**

We combine protein interaction data sets from multiple species, molecular sequences, and gene ontology to construct a set of high-confidence domain-domain interactions. First, we propose a new measure, the expected number of interactions for each pair of domains, to score domain interactions based on protein interaction data in one species and show that it has similar performance as the E-value defined by Riley *et al*. [1]. Our new measure is applied to the protein interaction data sets from yeast, worm, fruitfly and humans. Second, information on pairs of domains that coexist in known proteins and on pairs of domains with the same gene ontology function annotations are incorporated to construct a high-confidence set of domain-domain interactions using a Bayesian approach. Finally, we evaluate the set of domain-domain interactions by comparing predicted domain interactions with those defined in iPfam database [2,3] that were derived based on protein structures. The accuracy of predicted domain interactions are also confirmed by comparing with experimentally obtained domain interactions from *H. pylori *[4]. As a result, a total of 2,391 high-confidence domain interactions are obtained and these domain interactions are used to unravel detailed protein and domain interactions in several protein complexes.

**Conclusion:**

Our study shows that integration of multiple biological data sets based on the Bayesian approach provides a reliable framework to predict domain interactions. By integrating multiple data sources, the coverage and accuracy of predicted domain interactions can be significantly increased.

## Background

With the completion of genome sequences of many species, comparative analysis of these organisms becomes increasingly important in understanding the function and evolution of genes and proteins. Comparison of the genome sequences between worm and yeast has revealed that most of the core biological functions were carried out by orthologous proteins, and that the multi-cellular worm had more diverse proteins than the unicellular yeast [5]. In addition, more than 50 bacterial, archaeal, and eukaryotic genomes have been analyzed for protein function prediction, phylogenetic profiling of domains, and eukaryotic-signature domain organizations [6].

The development of high-throughput technologies such as yeast two-hybrid assays has produced large scale protein interaction data sets for several species, and significant efforts have been made to analyze them. By combining protein interaction data sets and orthology information on yeast protein sequences and a bacterial pathogen, Kelley *et al*. [7] and Sharan *et al*. [8] identified conserved protein interaction pathways and complexes. Further studies on conserved protein complexes and functional modules can be found in [9,10].

The basic units of proteins are domains and proteins interact with each other through their domains. Therefore, it is crucial to understand protein interactions at the level of the domains [11]. Several groups have developed methods to understand domain interactions based on protein interactions. Sprinzak and Margalit [12] selected domain interaction pairs based on the frequency of observed protein interactions that contain the pair of domains over its expect value. Deng *et al*. [13] developed a maximum likelihood estimation (MLE) method and an Expectation-Maximization (EM) algorithm to infer underlying domain interactions from protein interactions. Liu *et al*. [14] extended the MLE method to combine protein interactions from multiple species, and showed that the extension resulted in a higher accuracy in predicting protein interactions than using the yeast protein interactions alone. Liu *et al*. [14] also showed that, for a single species, the approach by Deng *et al*. [13] was comparable to that of Gomez *et al*. [15] and outperformed those of the Sprinzak and Margalit [12] and the Gomez *et al*. [16] for predicting protein interactions. More recently, Riley *et al*. [1] modified the Deng *et al*. [13] approach to be applicable to all the protein interactions in DIP [17,18] assuming no false positives and false negatives. Most importantly, they presented a new score for domain interactions, the E-score, defined as the log likelihood ratio of the observed interactions assuming the domain pairs interact over assuming the domain pairs do not interact. They showed that the E-score outperformed the Deng *et al*. [13] method in predicting domain interactions. Other approaches for predicting domain interactions using multiple data sources were developed in [19,20]. In this study, we focus on the integration of multiple data sources from multiple species to predict high-confidence domain interactions. First, we calculate the probability of domain interactions from four species: yeast [21-23], worm [24], fruitfly [25] and humans [26], respectively. Using these probabilities, we compute the expected number of interactions for each pair of domains within a species. Second, we investigate information on protein fusion and the domain functions. Third, a Bayesian approach is used to integrate those data sources to predict high-confidence domain interactions. These predictions help us to unravel the domain interactions in protein complexes and protein interactions. Our study differs from previous studies in several significant ways. Compared to Liu *et al*. and Ng *et al*. [14,19,20], our approach develop a new measure to score domain-domain interactions and validate it with experimentally derived domain interactions instead of using indirect ways such as validating re-inferred protein interactions. Compared to Riley *et al*. [1], protein fusion and Gene Ontology (GO) [27] functions are also integrated using a Bayesian approach. We show that the integration significantly increases the accuracy of predicted domain-domain interactions.

The paper is organized as follows. In the Methods section, we present the various data sources used in our analysis, followed by the methods for analyzing an integration of the different data sources. In the Results section, we present the results based on the various data sources separately, followed by the results based on integrated analysis. We evaluate our results by comparing with the domain-domain interactions in iPfam. Finally, we show limitations of our approach and further studies.

## Methods

### Data sources

In this study, we collect protein interactions and protein domain information from various databases for yeast, worm, fruitfly, and humans. Protein domain information is based on the Pfam-A domains [28]. Table 1 shows the number of proteins and protein interactions used in this study. Because only a subset of proteins contain Pfam-A domains, we use this subset along withtheir protein interactions in this study.

#### Protein interactions for yeast and worm

We download the protein interaction data sets for yeast and worm from the DIP database [17,18]. Each protein is associated with a DIP number, SWISSPROT ID, GI number, etc. We use the SWISSPROT accession numbers to associate domain information from the Pfam database [29] with the proteins in the DIP. We also use the GI numbers to obtain additional Pfam domain information from the National Center for Biotechnology Information [30]. For worm, the domain information collected using the GI numbers increases the number of protein interactions with domain information.

#### Protein interactions for fruitfly

We obtain the protein interaction data set for fruitfly from Giot *et al*. [25]. In this data set, protein names are identified by CG numbers. To obtain the relationship between proteins and domains, we associate the CG numbers with the SWISSPROT accession numbers by the protein table Integr8 in EMBL-EBI [31]. The compiled SWISSPROT accession numbers are used to extract protein-domain relationship from the Pfam database.

#### Protein interactions for human

We obtain the human protein interaction data set from the Human Protein Reference Database (HPRD) [26], which contains protein-protein interactions from individual small-scale experiments published in theliterature. The proteinsare identified by NP numbers. We associate the NP numbers in the HPRD with the SWISSPROT accession numbers using the protein table Integr8 in EMBL-EBI, and then extractprotein-domain relationship from the Pfam database.

#### Domain functions

We obtain domain functions, biological process, using the mapping table from Pfam to GO in the Gene Ontology webpage [27] and use the domains in the table to compile domain pairs with the same function.

#### Domain fusion

We use protein-domain information in Pfam-A to identify pairs of domains co-existing in one protein. The method is referred to as domain fusion in the rest of the paper.

#### Databases of domain interactions

We use two structure based domain interactions: iPfam [3] and Protein Quaternary Structure (PQS) [32] to estimate the reliability of predicted domain-domain interactions. iPfam contains 2,580 domain interactions(July 2004 version). The domain interactions in iPfam are obtained by calculating all bonds between all pairs of residues between domains based on the protein structures in Protein Data Bank (PDB). PQS provides probable quaternary states for structures based on PDB. In PQS, the analysis of determining biologically relevant interactions and crystal packing is attempted based on some known properties such as hydrophobicity, shape analysis, and the size of the solvent-accessible surface area (asa). Note that biologically relevant domain interactions and crystal contacts are not distinguished in iPfam. As domains in PQS are annotated by SCOP superfamily, we associate them with the Pfam domains using the mapping table in the SCOP webpage [33]. Finally, we obtain 36,439 domain interactions.

### Computational methods

In this subsection, we describe (1) the computational methods for calculating the probability of domain-domain interactions, (2) a new measure to evaluate the strength of domain-domain interactions, and (3) a Bayesian method for integrating different data sources to construct a high-confidence set of domain-domain interactions.

#### The maximum likelihood estimation for probabilities of domain-domain interactions

The maximum likelihood estimation method proposed by Deng *et al*. [13] has been shown to have good performance in estimating the probabilities of domain-domain interactions. We adopt this method in this study and briefly describe the method as follows.

The basic assumption of the MLE method is that two proteins interact if and only if at least one pair of domains from each of the two proteins interact. Given two proteins *P*_*i *_and *P*_*j*_, the probability that they interact is



where *P*_*ij *_= 1 if they interact and 0 otherwise, and *D*_*mn *_∈ _*ij *_denotes that domains *D*_*m *_and *D*_*n *_belong to proteins *P*_*i *_and *P*_*j*_, respectively, and *D*_*mn *_= 1 if domain *D*_*m *_interacts with domain *D*_*n*_. For an experiment in a species, the false positive rate (*fp*) is defined as the probability that two non-interacting proteins were observed to interact and the false negative rate (*fn*) is defined as the probability that two truly interacting proteins were not observed to interact in the experiment. Let *O*_*ij *_= 1 if the interaction between proteins *P*_*i *_and *P*_*j *_is observed and *O*_*ij *_= 0 otherwise. Thus, the probability for the observed protein interaction is

Pr(*O*_*ij *_= 1) = Pr(*P*_*ij *_= 1)(1 - *fn*) + (1 - Pr(*P*_*ij *_= l))*fp*.     (2)

The likelihood function-the probability of the whole interaction data set is

L=∏ij(Pr⁡(Oij=1))Oij(1−Pr⁡(Oij=1))1−Oij.     (3)
 MathType@MTEF@5@5@+=feaafiart1ev1aaatCvAUfKttLearuWrP9MDH5MBPbIqV92AaeXatLxBI9gBaebbnrfifHhDYfgasaacH8akY=wiFfYdH8Gipec8Eeeu0xXdbba9frFj0=OqFfea0dXdd9vqai=hGuQ8kuc9pgc9s8qqaq=dirpe0xb9q8qiLsFr0=vr0=vr0dc8meaabaqaciaacaGaaeqabaqabeGadaaakeaacqWGmbatcqGH9aqpdaqeqbqaaiabcIcaOiGbccfaqjabckhaYjabcIcaOiabd+eapnaaBaaaleaacqWGPbqAcqWGQbGAaeqaaOGaeyypa0JaeGymaeJaeiykaKIaeiykaKYaaWbaaSqabeaacqWGpbWtdaWgaaadbaGaemyAaKMaemOAaOgabeaaaaaaleaacqWGPbqAcqWGQbGAaeqaniabg+GivdGccqGGOaakcqaIXaqmcqGHsislcyGGqbaucqGGYbGCcqGGOaakcqWGpbWtdaWgaaWcbaGaemyAaKMaemOAaOgabeaakiabg2da9iabigdaXiabcMcaPiabcMcaPmaaCaaaleqabaGaeGymaeJaeyOeI0Iaem4ta80aaSbaaWqaaiabdMgaPjabdQgaQbqabaaaaOGaeiOla4IaaCzcaiaaxMaadaqadaqaaiabiodaZaGaayjkaiaawMcaaaaa@5C8C@

Our objective is to maximize the likelihood *L*, which can be represented as the function of *P*(*D*_*mn *_= 1) with fixed *fp *and *fn *by incorporating Equations 1, 2, and 3. *P*(*D*_*mn *_= 1) can be estimated by an expectation-maximization (EM) algorithm [13]. Deng *et al*. [13] presented a method to approximate the values of *fn *and *fp *based on the number of observed interactions. We combine this idea and the reliability of protein interaction data sets to approximate values of *fn *and *fp *in each species used in this study. The results are shown in Table 1. The details are presented in the additional file 1.

#### The expected number of occurrences of domain interactions

Deng *et al*. [13] used the estimated value of *P*(*D*_*mn *_= 1) to rank domain-domain interactions. One problem of the approach is that the estimated value of *P*(*D*_*mn *_= 1) is generally large if (1) each of the two domains appears only in one protein, (2) each of these two proteins contains only one domain, and (3) these two proteins interact. Another problem is that the value of *P*(*D*_*mn *_= 1) is generally small if (1) both domains appear in many proteins and (2) only a small proportion of these pairs of proteins having these two domains interact.

In order to overcome these problems, we score each domain pairs by the expected number of occurrences of domain interactions.

E(#*D*_*mn*_) = *N*_*mn *_Pr(*D*_*mn *_= 1),     (4)

where *N*_*mn *_is the number of protein pairs having domains *D*_*m *_and *D*_*n*_. Our intuition is that if a pair of domains are observed in multiple protein interactions, this pair of domains are more likely to interact. We use E() as a feature in our integrative model.

#### Domain fusion

In addition to the protein interaction data, we also incorporate information on domain fusion and domain function to build a set of high-confidence domain-domain interactions. Enright *et al*. [34] and Marcotte *et al*. [35] showed that two proteins are more likely to interact if they are fused into one protein in another species. This idea can be further extended to domains in that if two domains are fused in one protein in any species, they are more likely to interact. Thus, we search proteins having multiple Pfam-A domains and 9,615 Pfam-A domain pairs that co-exist in the same proteins are obtained. We define CE(*D*_*mn*_), where CE stands for Co-Existence, as the number of occurrences that domain *D*_*m *_and domain *D*_*n *_co-exist in the same proteins. It is expected that if CE(*D*_*mn*_) is larger, domain *D*_*m *_and domain *D*_*n *_are more likely to interact. We use CE() as a feature in our integrative model.

#### Domain functions

We obtain gene ontology terms of domains and find 57,907 domain pairs having the same GO terms in the category of the biological process. The gene ontology has a hierarchical structure (a directed acyclic graph), where the parents denote functions of more general terms and the offsprings represent functions of more specific terms. It is expected that two domains participating in the same GO function (biological process) are more likely to interact than they do in different functions. Moreover, two domains participating in a more specific function are more likely to interact than they do in a more general function. A more specific function generally covers a smaller number of domains. Assume that domain *D*_*m *_and domain *D*_*n *_have the same function *F*_*f*_. We define SG(*D*_*mn*_), where SG stands for the Same Gene ontology, as the number of domains having the function *F*_*f*_. We use SG() as a feature in our integrative model.

#### Integrating multiple data sources

The six information sources can be combined to construct a high-confidence set of domain-domain interactions. Several heuristic methods can be used for data integration. Here we consider three approaches: evidence counting, naïve Bayesian, and logistic regression.

For each pair of domains, six information sources for their interaction can be obtained from the analysis of the expected number of domain interactions derived from protein interactions of four species, the number of occurrences in the domain fusion, and the number of domains with the same GO annotation. We applied the aforementioned three computational methods to integrate these six biological evidences to predict domain interactions. The methods are described as follows.

##### Evidence counting

The number of evidences supporting domain interactions is used to score domain pairs for potential interactions. For a pair of domains *D*_*m *_and *D*_*n*_, we say that the interaction between *D*_*m *_and *D*_*n *_is supported by the yeast protein interactions if the expected number of occurrences of domain interactions is at least 1, i.e *E*(#*D*_*mn*_) ≥ 1. We count this as one evidence. A domain interaction can have a maximum of 4 evidences from yeast, worm, fruitfly and humans. Similarly, we say that the interaction between *D*_*m *_and *D*_*n *_is supported by the domain fusion if *CE*(*D*_*mn*_) ≥ 1, and by the domain functions if *SG*(*D*_*mn*_) ≥ 1. The number of evidences for a pair of domains ranges from 0 to 6.

##### Naïve Bayesian

The naïve Bayesian approach assumes the independence of data sources, and has been applied to the integration of multiple data sources for predicting protein interactions [36,37]. The basic idea is to calculate the likelihood ratio of each of the six evidences and then multiply these likelihood ratios. We define the set of observed interactions (Obs) as the interacting domain pairs in iPfam and the set of non-observed interactions (Nobs) as the domain pairs not presented in iPfam. The likelihood ratio for six data sources are calculated as follows. For each species, we split the values of E(#*D*_*mn*_) into 7 intervals. We call an interval as a bin, and this process as a binning process. Let *d *= E(#*D*_*mn*_) and *d *falls into the *t*-th bin. Let Pr(*d*|*Obs*) be the fraction of the observed interactions in the *t*-th bin and let Pr(*d*|*Nobs*) be the fraction of the non-observed interactions in the *t*-th bin. Then, the likelihood ratio for the *t*-th bin is Pr(*d*|*Obs*)/Pr(*d*|*Nobs*). Similarly, we bin the values of CE(*D*_*mn*_) and SG(*D*_*mn*_) and then calculate the likelihood ratio for each of them. Additional file 2 shows the likelihood ratios for each data source. Let *d*_1_,..., *d*_4 _be the values of E(#*D*_*mn*_) in yeast, worm, fruitfly, and humans, respectively, and let *d*_5 _and *d*_6 _be the values of CE(*D*_*mn*_) and SG(*D*_*mn*_), respectively. Then, the total likelihood ratio is

L(Dmn)=∏i=16Pr⁡(di|Obs)Pr⁡(di|Nobs).
 MathType@MTEF@5@5@+=feaafiart1ev1aaatCvAUfKttLearuWrP9MDH5MBPbIqV92AaeXatLxBI9gBaebbnrfifHhDYfgasaacH8akY=wiFfYdH8Gipec8Eeeu0xXdbba9frFj0=OqFfea0dXdd9vqai=hGuQ8kuc9pgc9s8qqaq=dirpe0xb9q8qiLsFr0=vr0=vr0dc8meaabaqaciaacaGaaeqabaqabeGadaaakeaacqqGmbatcqGGOaakcqWGebardaWgaaWcbaGaemyBa0MaemOBa4gabeaakiabcMcaPiabg2da9maarahabaWaaSaaaeaacyGGqbaucqGGYbGCcqGGOaakcqWGKbazdaWgaaWcbaGaemyAaKgabeaakiabcYha8jabd+eapjabdkgaIjabdohaZjabcMcaPaqaaiGbccfaqjabckhaYjabcIcaOiabdsgaKnaaBaaaleaacqWGPbqAaeqaaOGaeiiFaWNaemOta4Kaem4Ba8MaemOyaiMaem4CamNaeiykaKcaaaWcbaGaemyAaKMaeyypa0JaeGymaedabaGaeGOnaydaniabg+GivdGccqGGUaGlaaa@568B@

##### Logistic regression

Let *E*_*y*_(#*D*_*mn*_), *E*_*w*_(#*D*_*mn*_), *E*_*f*_(#*D*_*mn*_), and *E*_*h*_(#*D*_*mn*_) denote the expected number of occurrences of the domain interactions in yeast, worm, fruitfly and humans, respectively. Let I(*d*) be the indicator function: I(*d*) = 1 if *d *≥ 1 and 0, otherwise. Let *EV*(*D*_*mn*_) be the number of evidences from the *evidence counting *method. We use the following model,

log⁡Pr⁡(Dmn=1)1−Pr⁡(Dmn=1)=α+β1Ey(#Dmn)+β2Ew(#Dmn)+β3Ef(#Dmn)+β4Eh(#Dmn)+β5I(CE(Dmn))+β6I(SG(Dmn))+β7EV(Dmn).
 MathType@MTEF@5@5@+=feaafiart1ev1aaatCvAUfKttLearuWrP9MDH5MBPbIqV92AaeXatLxBI9gBaebbnrfifHhDYfgasaacH8akY=wiFfYdH8Gipec8Eeeu0xXdbba9frFj0=OqFfea0dXdd9vqai=hGuQ8kuc9pgc9s8qqaq=dirpe0xb9q8qiLsFr0=vr0=vr0dc8meaabaqaciaacaGaaeqabaqabeGadaaakeaafaqaaeGadaaabaGagiiBaWMaei4Ba8Maei4zaC2aaSaaaeaacyGGqbaucqGGYbGCcqGGOaakcqWGebardaWgaaWcbaGaemyBa0MaemOBa4gabeaakiabg2da9iabigdaXiabcMcaPaqaaiabigdaXiabgkHiTiGbccfaqjabckhaYjabcIcaOiabdseaenaaBaaaleaacqWGTbqBcqWGUbGBaeqaaOGaeyypa0JaeGymaeJaeiykaKcaaaqaaiabg2da9aqaaGGaciab=f7aHjabgUcaRiab=j7aInaaBaaaleaacqaIXaqmaeqaaOGaemyrau0aaSbaaSqaaiabdMha5bqabaGccqGGOaakcqGGJaWicqWGebardaWgaaWcbaGaemyBa0MaemOBa4gabeaakiabcMcaPiabgUcaRiab=j7aInaaBaaaleaacqaIYaGmaeqaaOGaemyrau0aaSbaaSqaaiabdEha3bqabaGccqGGOaakcqGGJaWicqWGebardaWgaaWcbaGaemyBa0MaemOBa4gabeaakiabcMcaPiabgUcaRiab=j7aInaaBaaaleaacqaIZaWmaeqaaOGaemyrau0aaSbaaSqaaiabdAgaMbqabaGccqGGOaakcqGGJaWicqWGebardaWgaaWcbaGaemyBa0MaemOBa4gabeaakiabcMcaPiabgUcaRiab=j7aInaaBaaaleaacqaI0aanaeqaaOGaemyrau0aaSbaaSqaaiabdIgaObqabaGccqGGOaakcqGGJaWicqWGebardaWgaaWcbaGaemyBa0MaemOBa4gabeaakiabcMcaPaqaaaqaaaqaaiabgUcaRiab=j7aInaaBaaaleaacqaI1aqnaeqaaOGaemysaKKaeiikaGIaem4qamKaemyrauKaeiikaGIaemiraq0aaSbaaSqaaiabd2gaTjabd6gaUbqabaGccqGGPaqkcqGGPaqkcqGHRaWkcqWFYoGydaWgaaWcbaGaeGOnaydabeaakiabdMeajjabcIcaOiabdofatjabdEeahjabcIcaOiabdseaenaaBaaaleaacqWGTbqBcqWGUbGBaeqaaOGaeiykaKIaeiykaKIaey4kaSIae8NSdi2aaSbaaSqaaiabiEda3aqabaGccqWGfbqrcqWGwbGvcqGGOaakcqWGebardaWgaaWcbaGaemyBa0MaemOBa4gabeaakiabcMcaPiabc6caUaaaaaa@A726@

#### Validating the predicted domain interactions

To evaluate the reliability of the predicted domain interactions, we compare them with the domain interactions in iPfam. The interactions in iPfam are treated as the observed interactions. Although many domain interactions are not included in the database, a good score function for domain interactions should include a higher fraction of observed interactions in the highest ranked predictions than a random scoring function. Therefore, for a given scoring range, the fraction of the observed interactions among all domain pairs having scores within the range is calculated. We also calculate the ratio of this fraction over that from a random scoring function and refer to it as the fold value. For a good score function, the fold value should increase with the score.

Another method to evaluate the reliability of predicted domain interactions is using the Receiver Operating Characteristic (ROC) curve representing the relationship between false positive rate (FPR) and sensitivity (SN). As we mentioned before, we use domain pairs in iPfam as the observed interactions and domain pairs not in iPfam as the non-observed interactions. Because this gives too many non-observed interactions (1,536,555), we randomly remove domain pairs without any evidence and finally obtain 84,385 domain pairs, about twice of the number of domain pairs with at least one evidence, for the non-observed set. For a given threshold value t, domain pairs with score larger than t are predicted as interacting and others as non-interacting. The results can be represented as

The FPR and SN are defined as

FPR=FPFP+TN,SN=TPTP+FN.
 MathType@MTEF@5@5@+=feaafiart1ev1aaatCvAUfKttLearuWrP9MDH5MBPbIqV92AaeXatLxBI9gBaebbnrfifHhDYfgasaacH8akY=wiFfYdH8Gipec8Eeeu0xXdbba9frFj0=OqFfea0dXdd9vqai=hGuQ8kuc9pgc9s8qqaq=dirpe0xb9q8qiLsFr0=vr0=vr0dc8meaabaqaciaacaGaaeqabaqabeGadaaakeaafaqabeqacaaabaGaemOrayKaemiuaaLaemOuaiLaeyypa0ZaaSaaaeaacqWGgbGrcqWGqbauaeaacqWGgbGrcqWGqbaucqGHRaWkcqWGubavcqWGobGtaaGaeiilaWcabaGaem4uamLaemOta4Kaeyypa0ZaaSaaaeaacqWGubavcqWGqbauaeaacqWGubavcqWGqbaucqGHRaWkcqWGgbGrcqWGobGtaaGaeiOla4caaaaa@45EC@

We use five-fold cross-validation to compare the performance. We use a subset of iPfam domain interactions for training to calculate the likelihood ratio of the Bayesian approach and the coefficients of the logistic regression. The remaining iPfam domain interactions are used for testing.

## Results

### Conserved domain interactions across multiple species

**Figure 1:**
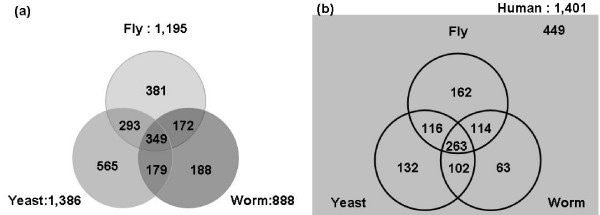
A Venn diagram for the numbers of domains in yeast, worm, fruitfly, and humans. (a) The numbers of domains in yeast, worm, and fruitfly. (b) The numbers of domains between humans and the other three species.

We first show that domain interactions inferred from multiple species are reliable. The four species share many domains. Table 1 shows the number of proteins, the numbers of protein-protein interactions, and the numbers of domains in each species. Figure 1 shows the numbers of domains overlapped among the different species. Most domains appear in more than one species. For example, 953 out of 1,386 domains in yeast (69%) are found in at least one of the other three species. Similarly, 763 out of 888 domains in worm (86%) are found in other species. For fruitfly and humans, 82% and 68% are found in other species, respectively.

**Figure 2:**
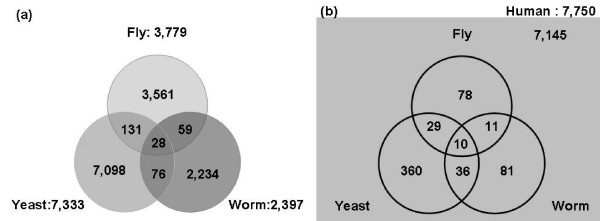
A Venn diagram for the numbers of predicted domain-domain interactions in yeast, worm, fruitfly, and humans. (a) The numbers of predicted domain-domain interactions in yeast, worm, and fruitfly. (b) The numbers of predicted domain-domain interactions between humans and the other three species.

We apply the MLE method to calculate probabilities of domain interactions. The numbers of domain interactions obtained (probability>0) for yeast, worm, fruitfly, and humans are 7,333, 2,397, 3,779, and 7,750, respectively. Figure 2 shows the numbers of predicted domain interactions among four species together with the overlaps. 812 (4.0%) out of a total of 20,332 predicted domain interactions from the four species are presented in at least two species, which we call predicted conserved domain interactions. Although this fraction is relatively small, we find that this fraction is still three times higher than that of random interactions [See additional file 3]. This result is consistent with other studies [7,38], which found only a small percentage of conserved protein interactions across several species. We compare these 812 domain interactions with iPfam. Table 2 shows that, surprisingly, 18.2% of the 812 conserved domain interactions are found in iPfam, compared to only 3.0% for all of the predicted 20,332 domain interactions. Furthermore, 50% of the domain interactions presented in all four species belong to iPfam. The results suggest that the predicted conserved domain interactions obtained from at least two species are very reliable. Similar results are obtained (Table 2) by comparing the predicted conserved domain interactions with domain interactions obtained from the Protein Quaternary Structure (PQS) database [32]. The list of predicted conserved domain interactions from at least three species is presented in additional file 4.

### Contributions of each data source to the accuracy of predicted domain interactions

**Figure 3:**
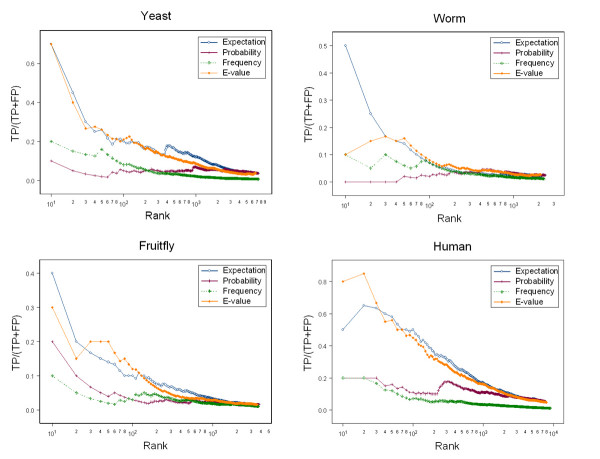
The relationship between rank and true positive rate (TP/(TP+FP)) compared to the iPfam for four species based on four score functions. "Expectation" ranks domain pairs according to the expected number of occurrences of domain pairs in protein interactions; "Probability" ranks domain pairs according to the estimated probability of interactions from the MLE method; "Frequency" ranks domain pairs according to the number of protein interactions having domain pair; "E-value" ranks domain pairs according to the E-value defined in [1].

We first evaluate the contributions of each of the six information sources to the accuracy of predicted domain-domain interactions by comparing with the domain interactions in iPfam. To score domain interactions based on protein interactions, three measures are considered. The first measure is based on the estimated value of the probability of domain interactions (*P*(*D*_*mn *_= 1)). The second is the number of times the domain pairs occur in protein pairs (*N*_*mn*_). The last is the multiplication of the first two, *N*_*mn*_*P*(*D*_*mn *_= 1). These measures are referred as *probability*, *frequency*, and *expectation*, respectively. We also compare with another measure defined as *E-value *by [1]. The performance of each score function is evaluated by the true positive rate (TP/(TP+FP)) among the top r ranked domain pairs. For each score function and a rank value r, domain pairs with the top r ranked scores are predicted as interacting. The predicted domain interactions are compared with domain interactions in iPfam. Figure 3 shows the relationship between the true positive rate and the rank r based on the four different score functions. For given r, the fractions of observed interactions among the top r ranked domain pairs based on *expectation *and *E-value *are higher than that based on *probability *and *frequency*. Figure 3 indicates that the scores based on *expectation *and *E-value *have similar performance and outperform the other two scores in evaluating domain interactions. As another way of comparison, we also draw ROC curves based on the four score functions and they are given in additional file 5. The relative performance of the four score measures based on ROC curves is similar as above.

We next consider the relationship between domain fusion and domain interactions. Similar ideas have been applied to *E. coli *and *S. cerevisiae *to infer protein interactions [34,35]. From Pfam, we collect 9,615 Pfam-A domain pairs that co-exist in the same proteins, among which 1,141 overlap with iPfam (Table 3). 859 domain pairs found through domain fusion are found to interact within at least one species based on protein interaction data, among which 283 (32.9%) overlap with iPfam. The results suggest that the co-existence of domain pairs is a reliable evidence for domain interactions and combining multiple evidences reduces the number of false positives.

We also incorporate information on domain pairs with the same GO annotations. It is known that proteins having similar functions are more likely to interact [38,39]. In fact, the observation is true for domains as well. We find 57,907 domain pairs having the same GO terms in the category of biological process. 1,031 domain pairs are also found in predicted domain interactions based on protein interaction data, among which 234 (22.7%) domain interactions are found in iPfam (Table 3).

### Integration of multiple biological data sources

We integrate six data sources using different methods described in the Methods section, and compare the performance using a five-fold cross-validation. We first show the improvement of integrating multiple biological data sources. Table 3 shows the percentages of overlaps between iPfam and the predicted domain interactions with multiple evidences. The results indicate that one single evidence is not sufficient for predicting domain interactions as only 8.8% of these domain interactions overlap with iPfam. However, the percentage of overlaps increases to 50.5% for domain interactions with two or more evidences. As the number of evidences increases, the predictions are more accurate but, the number of predictions decreases at the same time. Only 58 predicted domain interactions have four or more evidences and 43 out of 58 (= 74.1%) belong to iPfam.

Table 4 shows the percentages of overlaps between iPfam and the predicted domain interactions based on the Bayesian approach. The fraction of domain pairs overlapped with iPfam increases as the likelihood ratio score increases. 80.0% of the 420 domain pairs with likelihood ratio scores greater than 50 are found in iPfam, a 471-fold increase over that of random domain pairs. Comparing Table 3 with Table 4, we conclude that the likelihood ratio score significantly increases the number of high-confidence domain interaction pairs.

**Figure 4:**
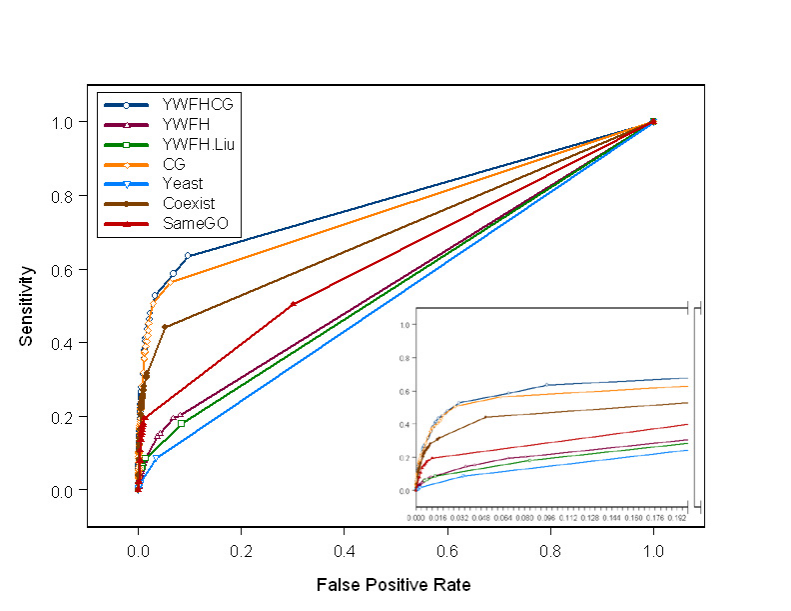
The relationship between false positive rate and sensitivity for predicting domain interactions using the Bayesian method with different data sources. The letters Y, W, F, H, C, and G indicate domain interactions based on yeast, worm, fruitfly, humans, co-existence, and same GO function, respectively. YWFH.Liu shows the result of predicted domain interactions using the extended MLE method defined in Liu *et al*. [14] with protein interactions of yeast, worm, fruitfly, and humans.

Figure 4 shows the ROC curves of the Bayesian method using multiple data sources. Combining all six data sources gives the highest accuracy. It also shows that adding the domain-fusion and domain function information significantly improves the performance of the prediction. In addition, we compare the naïve Bayesian approach with the method by Liu *et al*. [14] where they multiplied the likelihoods of the observed protein interactions from four species to achieve one likelihood function. Figure 4 shows the ROC curves of the two approaches by using the protein interaction data from the four species. In both approaches, the *expectation *score of domain interactions is used. Although both approaches have similar performance, one advantage of the Bayesian approach is that other information such as domain fusion and domain function can easily be incorporated.

**Figure 5:**
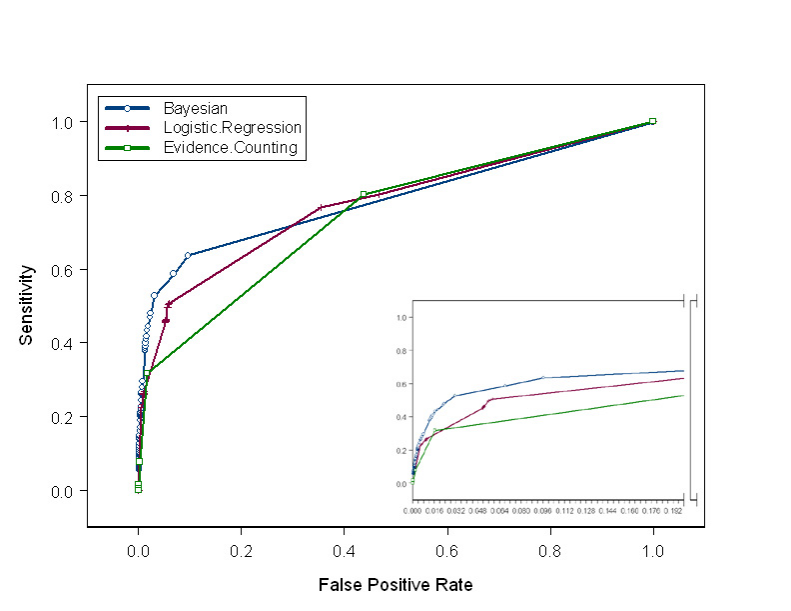
The relationship between false positive rate and sensitivity for predicting domain interactions using different methods : evidence counting, logistic regression, and naive Bayesian.

We compare the power of the three methods for predicting domain interactions: evidence counting, naïve Bayesian, and logistic regression. Figure 5 shows the ROC curves for the three methods. It is clear that the Bayesian approach outperforms the other two. The evidence counting method does not consider the quality of each data sources, and the logistic regression method is limited by many missing values. Finally, we select a set of 2,391 high-confidence domain interactions having the likelihood ratio value at least 4, among which more than half (51.9%) are found in the iPfam. This set covers 48.1% of the data in iPfam with a false positive rate of 2.3%. We list the top 10 predicted domain interactions that are not found in iPfam (July 2004 version) in Table 5. Among them, three were later included in the updated version of iPfam (Oct. 2005 version), showing the reliability of the high-confidence domain interactions. The list of the high-confidence domain interactions is shown in additional file 6 and likelihood ratio values of 25,352 domain pairs are given in additional file 7. In these tables, the domain pairs are sorted based on the Bayesian approach. The rankings by the three methods, the Bayesian approach, the logistic regression, and the evidence counting, are also presented to show the similarity of three methods. We test the differences of the rankings of the 25,352 domain pairs by three methods using the Wilcox rank sum test based on the null hypothesis of no difference between rankings. All three p-values are around 0.5, showing that the null hypothesis cannot be rejected. However, it does not indicate that the rankings by three approaches are similar. The ROC curves in Figure 5 show that the top ranked domain pairs by three methods are different.

### Comparison with domain interactions in *H. pylori*

Rain *et al*. [4] reported a protein-protein interaction data set for *H. pylori *using yeast two hybrid assays. This data set provides the ranges of sequences of the prey proteins interacting with the bait proteins. We map these ranges in the preys to the Pfam-A domains when the overlap between them is larger than 50% of the Pfam domains. As we do not have such information for the baits, we assume that all domains in the baits interact with the specific site of the preys. We obtain a total of 1,101 interactions between Pfam-A domains. Note that the domain interactions from *H. pylori *may contain false positives as the interacting domains in the baits are not known. We compare our predicted domain interactions from the six data sources using the Bayesian approach with the experimentally derived domain interactions from *H. pylori*. For comparison, we use a subset of the predicted domain interactions with domains involved in domain interactions in *H. pylori*. Additional file 8 shows the percentages of overlaps between the domain interactions from *H. pylori *and the predicted domain interactions. The fraction of domain pairs overlapped with the domain interactions in *H. pylori *increases as the likelihood ratio score increases, confirming the accuracy of the predicted domain interactions.

We also study our scoring algorithm using the *H. pylori *database. We infer domain interactions from *H. pylori *protein interactions using four scoring functions and compare the predicted domain interactions with the domain interactions from *H. pylori*. The number of domains in *H. pylori *is 848 and 848*849/2 = 359,976 are potential interacting pairs. From the *Expectation *scoring function, we obtain 1,150 predicted domain interactions (larger than zero). Among them, 750 predicted domain interactions overlap with the 1,011 domain interactions in *H. pylori*. Additional file 9 shows that true positive rate is around 0.8 in 1,150 ranked domain interactions, showing the accuracy of the scoring functions.

### Domain interactions in yeast complexes

**Figure 6:**
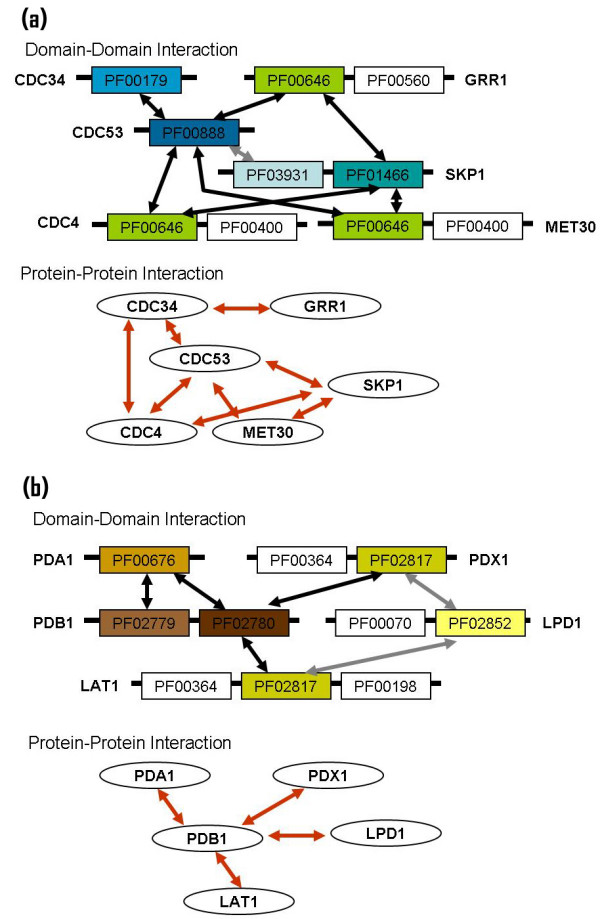
Two examples of yeast complexes with predicted domain-domain interactions and MIPS physical protein interactions. The black arrows are predicted DDIs, the grey arrows are DDIs in iPfam, and the red arrows are PPIs from DIP. (a) SCF (Skp1-Cdc53-F-box protein) complexes. Cdc53 controls G1/S transition. Cdc34 is E2 ubiquitin-conjugating enzyme. Skp1 is kinetochore protein complex Cbf3, subunit D. Cdc4, Met30, and Grr1 are the F-box proteins. (b) Pyruvate dehydrogenase complexes. Pdb1 is pyruvate dehydrogenase (lipoamide) beta chain precursor, Pda1 is pyruvate dehydrogenase (lipoamide) alpha chain precursor, Lpd1 is dihydrolipoamide dehydrogenase precursor, Pdx1 is pyruvate dehydrogenase complex protein X, and Lat1 is dihydrolipoamide S-acetyltransferase. For details, see the main text.

We apply the set of high-confidence domain interactions to examine the detailed protein and domain interactions in yeast complexes [21]. Figure 6 shows two examples of protein complexes. Figure 6(a) is the SCF (Skp1-Cdc53-F-box protein) complex. SCF is a multi-protein complex with Cdc53, Skp1, and at least three independent F-box proteins, Cdc4, Met30, and Grr1 [40]. This complex acts as a ubiquitin ligase catalyzing the final ubiquitin-transfer reaction in the destruction of G1/S-cyclins. Our prediction of domain interaction is consistent with the literature in that only domain PF00646 (F-box domain) of F-box proteins such as Cdc4, Met30, and Grr1 interact with domain PF01466 of protein Skp1. Domain PF00400 (Leucine Rich Repeat domain) and domain PF00560 (WD domain, G-beta repeat) do not participate in protein-protein interactions. Patton *et al*. [40] suggested that Cdc53 is a scaffold protein for Cdc34 and Skp1 by showing that it has independent binding sites for Cdc34 and Skp1. Our result also shows that the domain PF00888 in the protein Cdc53 has interaction with both the domain PF00179 of the protein Cdc34 and the domain PF01466 of the protein Skp1.

Figure 6(b) shows a Pyruvate dehydrogenase (PDH) complex. This complex converts pyruvate to acetyl CoA. The interaction between protein Lat1 and protein Pdb1 is mainly due to the interaction between domain PF02817 and domain PF02780. Domain PF02817 is an E3 binding domain, and PF02780 is the C-terminal domain of transketolase, which has been proposed as a regulatory molecule binding site. The interaction between protein Lap1 and protein Lpd1 occurs through the interaction of domain PF02817 and domain PF02852, which is the Pyridine nucleotide-disulphide oxidoreductase, dimerisation domain.

## Discussion

The basic units of proteins are domains. If two proteins interact, at least one pair of domains from each of the two proteins interact. However, current biotechnologies such as the yeast-two-hybrid system can only detect protein interactions and it is tedious and labor intensive to derive domain interactions. The prediction of domain interactions based on protein interactions from one species has been formulated as a missing value problem and an EM algorithm has been developed to achieve this objective [13]. The method has been modified to integrate protein interaction data sets from multiple species and the results have been improved [1,14]. In this study, we further explore the problem of domain-domain interactions from multiple data sources including protein interactions from four species; yeast, worm, fruitfly, and humans, as well as domain fusion and domain function information. We first provide a score function, the expected number of domain-domain interactions in the observed interactions, to infer the reliability of domain interactions. By comparing with domain interactions in iPfam, we show that the new score outperforms the score of Deng *et al*. [13] for predicting domain interactions. The true positive rate among highly ranked domain interactions predicted from the new score is higher than that from Deng *et al*. [13]. We further show that, by including the domain fusion and gene ontology information, the accuracy of the predicted domain interactions can be significantly increased. We also show that the simple naïve Bayesian approach works well to combine multiple biological information for predicting high-confidence domain interactions. There are several limitations of this study. First, we did not include all the interaction data from all the species as Riley *et al*. [1] did. The reason is that the size of data in other species is much smaller than those in the four species. Second, the protein interaction data sets used in this study are incomplete and contain many false positives. Additional file 1 shows the ROC curves of the prediction results using various values of false positive (fp) and false negative (fn). In particular, we compared the result based on the fp and fn values presented in Table 1 with the result based on fp = fn = 0 used in Riley *et al*. [1]. Depending on species, the former approach is sometimes better than or similar to the latter approach, and sometimes is worse. Third, although we have shown that the naïve Bayesian approach outperforms the evidence counting and the logistic regression methods, there is room to improve the prediction by considering the correlations between data sources.

## Conclusion

We have shown that the likelihood ratio score provides a mean for evaluating the reliability of domain interactions. Based on the likelihood ratio score, we have derived a set of high-confidence domain interactions. This set has important implication in understanding protein functions at the domain level as well as in understanding protein interactions.

## Abbreviations

MLE – Maximum Likelihood Estimation

EM – Expectation Maximization

HPRD – Human Protein Reference Database

GO – Gene Ontology

ROC – Receiver Operating Characteristic

FPR – False Positive Rate

SN – Sensitivity

PQS – Protein Quaternary Structure

## Authors' contributions

HL developed and implemented methods of inferring domain interactions by combinig multiple biological data sets, collected biological data sets, and drafted the manuscript. MD provided the program for expectation-maximization algorithm to infer domain interactions from protein interactions and helped the data collection. FS and TC initiated and directed this research and helped in writing the manuscript.

## Supplementary Material

Additional file 1 **False positive (*fp*) and false negative (*fn*) of the observed protein interactions**. It contains equations to calculate *fp *and *fn *values for the protein interactions used in the study and effects of various *fp *and *fn *values to the inference the domain interactions. Click here for file

Additional file 2 **The likelihood ratio of six data sources**. The values for domain interactions inferred from six data sources are binned into discrete intervals and the likelihood ratio is calculated. Click here for file

Additional file 3 **Comparison with predicted conserved domain interactions and random interactions**. Table S2 shows the significance of the number of predicted conserved domain interactions compared to the random interactions. Click here for file

Additional file 4 **List of conserved domain interactions predicted from protein interactions of at least three species**. These conserved domain interaction have 31% of overlaps with domain interactions in iPfam. Click here for file

Additional file 5 **ROC curves of predicted domain interactions using yeast, worm, fruitfly and humans**. Figure S2 shows the comparison of performances of score functions to predict domain interactions for four species. Click here for file

Additional file 6 **The 2,391 high-confidence domain interactions from the Bayesian approach**. Domain pairs are sorted by the rank based on the Bayesian approach. Rankings by evidence counting (EV) and Logistic Regression (LR) are presented with the number of evidences and the probability by LR. Click here for file

Additional file 7 **The likelihood ratio of all domain interactions**. Domain pairs with larger than zero likelihood ratio are sorted by the rank based on the Bayesian approach. Rankings by evidence counting (EV) and Logistic Regression (LR) are presented with the number of evidences and the probability by LR. Click here for file

Additional file 8 **The likelihood ratio values of predicted domain interaction, the numbers of predicted domain interactions, and the overlap with domain interactions from H. pylori**. We used 1,101 domain interactions in H. pylori involving 206 domains. Numbers in the first column indicate the likelihood ratio values for the domain interactions, and the second column is the number of interactions having the corresponding likelihood ratio values. "Fold" indicates the ratio of the fraction over expected value. (5.2%). Click here for file

Additional file 9 **A ROC curve of predicted domain interactions using H. pylori**. Figure S3 shows the comparison of performances of score functions to predict domain interactions for *H. pylori*. Click here for file

## Figures and Tables

**Table 1:** Data sets. The characteristics of protein interaction data sets for yeast, worm, fruitfly and humans, the corresponding-domain information, and the values of *fn *and *fp *used in the analysis. Only protein pairs with both proteins containing Pfam-A domains are included in the protein interaction data sets, and proteins in those protein interactions are counted. The numbers in the parenthesis are the total number of available protein interactions.

	Yeast	Worm	Fruitfly	Humans
Proteins	2,568	1,580	2,444	3,493
Protein-protein interactions	7,985 (15,461)	2,193 (4,030)	3,944 (20,429)	10,906 (15,274)
Domains	1,386	888	1,195	1,401
False Negative (fn)	0.25	0.67	0.61	0.25
False Positive (fp)	0.0009	0.0007	0.0005	0.0007

**Table 2:** The numbers of predicted domain interactions using protein interactions. The predicted domain interactions classified by the number of species (1,2,3 and 4) and their overlaps with iPfam and PQS.

Species	1	2	3	4	All
Predicted domain interactions	19,520	707	95	10	20,332
Overlap with iPfam (Ratio)	468 (2.4%)	115 (16.2%)	28 (29.5%)	5 (50%)	616 (3.0%)
Overlap with PQS (Ratio)	883 (4.5%)	147 (20.8%)	31 (32.6%)	4 (40%)	1,065 (5.2%)

**Table 3:** The numbers of predicted domain interactions using domain fusion, domain function, and combining six data sets. The predicted domain interactions, the number of evidences, and the overlaps with iPfam. Numbers in the first column indicate the number of evidences for the domain interactions, and the second column is the number of interactions having the corresponding evidences. "PPI" represents the protein interaction data sets. "Fraction" indicates the fraction of domain interactions in iPfam in a given set. "Fold" indicates the ratio of the fraction over expected value (0.17%).

Evidence	Interactions	Overlap with iPfam	Fraction	Fold
Random domain pairs	1,539,135	2,580	0.17%	-
Domain fusion	9,615	1,141	11.8%	69
Domain fusion & PPI	859	283	32.9%	194
Same GO terms	57,907	1,302	0.8%	13
Same GO terms & PPI	1,031	234	22.7%	134
≥ 1	23,606	2,071	8.8%	52
≥ 2	1,624	820	50.5%	297
≥ 3	307	200	65.1%	383
≥ 4	58	43	74.1%	436
≥ 5	13	10	76.9%	452
= 6	0	-	-	-

**Table 4:** The likelihood ratio values of predicted domain interactions. The likelihood ratio values of predicted domain interaction, the numbers of predicted domain interactions, and the overlap with iPfam. Numbers in the first column indicate the likelihood ratio values for the domain interactions, and the second column is the number of interactions having the corresponding likelihood ratio values.

Likelihood ratio values	Interactions	Overlap with iPfam	Fraction	Fold
Random domain pairs	1,539,135	2,580	0.17%	-

> 0	25,352	2,080	8.2%	48
≥ 1	6,386	1,641	25.7%	151
≥ 4	2,391	1,241	51.9%	305
≥ 6	2,044	1,142	55.9%	329
≥ 11	1,683	1,011	60.1%	353
≥ 21	886	634	71.6%	421
≥ 51	420	336	80.0%	471

**Table 5:** The ten highest ranked domain-domain interactions. The ten highest ranked domain-domain interactions from the Bayesian approach which are not in iPfam. iPfam_2005 represents domain interactions found in updated version of iPfam (Oct 2005 version).

Domain 1		Domain 2		iPfam_2005
Pfam ID	Accession	Pfam ID	Accession	
WD40	PF00400	Pkinase	PF00069	
zf-C2H2	PF00096	Pkinase	PF00069	
zf-C3HC4	PF00097	zf-C3HC4	PF00097	
F-box	PF00646	Skp1_POZ	PF03931	
zf-C4	PF00105	Hormone_recep	PF00104	x
SMC_hinge	PF06470	SMC_N	PF02463	x
Cation_ATPase_N	PF00690	Cation_ATPase_C	PF00689	
MutS_V	PF00488	MutS_I	PF01624	
Cadherin	PF00028	Cadherin_C	PF01049	
dsrm	PF00035	dsrm	PF00035	x

**Table 6:** 

	Prediction	
	Interacting	Non-interacting
Observed	TP	FN
Non-observed	FP	TN
