# Supplementary material for: An integrated approach to the prediction of domain-domain interactions
Source: BMC Bioinformatics. 2006 May 25;7:269. doi: 10.1186/1471-2105-7-269 (PMC1481624; doi:10.1186/1471-2105-7-269)
Supplement: Additional file 1 — False positive (fp) and false negative (fn) of the observed protein interactions. It contains equations to calculate fp and fn values for the protein interactions used in the study and effects of various fp and fn values to the inference the domain interactions. [file 1471-2105-7-269-S1.pdf]

False positive (fp) and false negative (fn)

We estimated fp and fn with the similar equation in Deng et al. [1]. The difference is the calculation of $Pr(O_{ij} = 1, P_{ij} = 1)$. Lee et al. [2] assessed the reliability of DIP protein interactions for yeast as 0.6, which represents $Pr(P_{ij} = 1|O_{ij} = 1)$. We modified the equations for fp and fn in Deng et al. [1] by using $P(O_{ij} = 1) * 0.6$ for $Pr(O_{ij} = 1, P_{ij} = 1)$. The average numbers of interaction partners in the experimental protein-protein interaction sets are 5.2, 0.4, 3.2, and 5.2 for yeast, worm, fruitfly, and humans, respectively. If we consider that the protein interaction sets are incomplete, especially for worm, and protein interactions are relatively well studied in yeast, we can assume that each protein has on average of at least five interaction partners. Hazbun and Fields [3] estimated that each protein interacts with about t = 5 to 50 proteins. We assume that each protein interacts with other proteins about t=5 times and we use it to estimate the total number of real interaction pairs. In addition, we also showed the results using t=50 in Figure S1. For example of yeast,

$$
\begin{aligned}
fn &= Pr(O_{ij} = 0|P_{ij} = 1) \\
&= 1.0 - \frac{Pr(O_{ij} = 1, P_{ij} = 1)}{Pr(P_{ij} = 1)} \\
&= 1.0 - \frac{Pr(O_{ij} = 1)Pr(P_{ij} = 1|O_{ij} = 1)}{Pr(P_{ij} = 1)} \\
&= 1.0 - \frac{number\ of\ observed\ interaction\ pairs}{number\ of\ real\ interaction\ pairs} * 0.6 \\
&= 1.0 - \frac{7,985}{t * N/2} * 0.6 \\
&= 1.0 - \frac{7,985}{5 * 2,568/2} * 0.6 \\
&= 0.25
\end{aligned}
$$

$$
\begin{aligned}
fp &= Pr(O_{ij} = 1|P_{ij} = 0) \\
&= \frac{Pr(O_{ij} = 1, P_{ij} = 0)}{Pr(P_{ij} = 0)} \\
&= \frac{Pr(O_{ij} = 1)Pr(P_{ij} = 0|O_{ij} = 1)}{Pr(P_{ij} = 0)} \\
&= \frac{number\ of\ observed\ interaction\ pairs}{total\ protein\ pairs - number\ of\ observed\ interaction\ pairs} * 0.4 \\
&= \frac{7,985}{N * (N+1)/2 - t * N/2} * 0.4 \\
&= \frac{7,985}{2,568 * (2,568+1)/2 - 5 * 2,568/2} * 0.4 \\
&= 0.0009
\end{aligned}
$$

where N is number of proteins in the DIP database with PFAM-A domain information. To assess the reliability of observed protein interactions using the method in Lee et al. [2], we need the gold standard protein interactions for each species. For yeast, the MIPS protein interaction set is used as a gold standard.

However, other three species do not have gold standard protein interaction sets. Therefore, we approximated the reliability of protein interaction sets of other species used in this study as the same with that of yeast.

We study the influence on the prediction accuracy of using the fp and fn values estimated in the above by comparing the performance with fp and fn estimated with t=50. Figure S1 shows the ROC curve compared to the iPfam based on the "Expectation" score function with different fp and fn values. The performance decreases more with fp and fn values estimated with t=50 than those with t=5. We also infer domain interactions using fp=fn=0. For yeast, the performance with fp and fn values estimated with t=5 outperforms that with fp=fn=0. However, for worm, fp=fn=0 gives better result. For fruitfly and humans, they have similar performance.

Figure S1

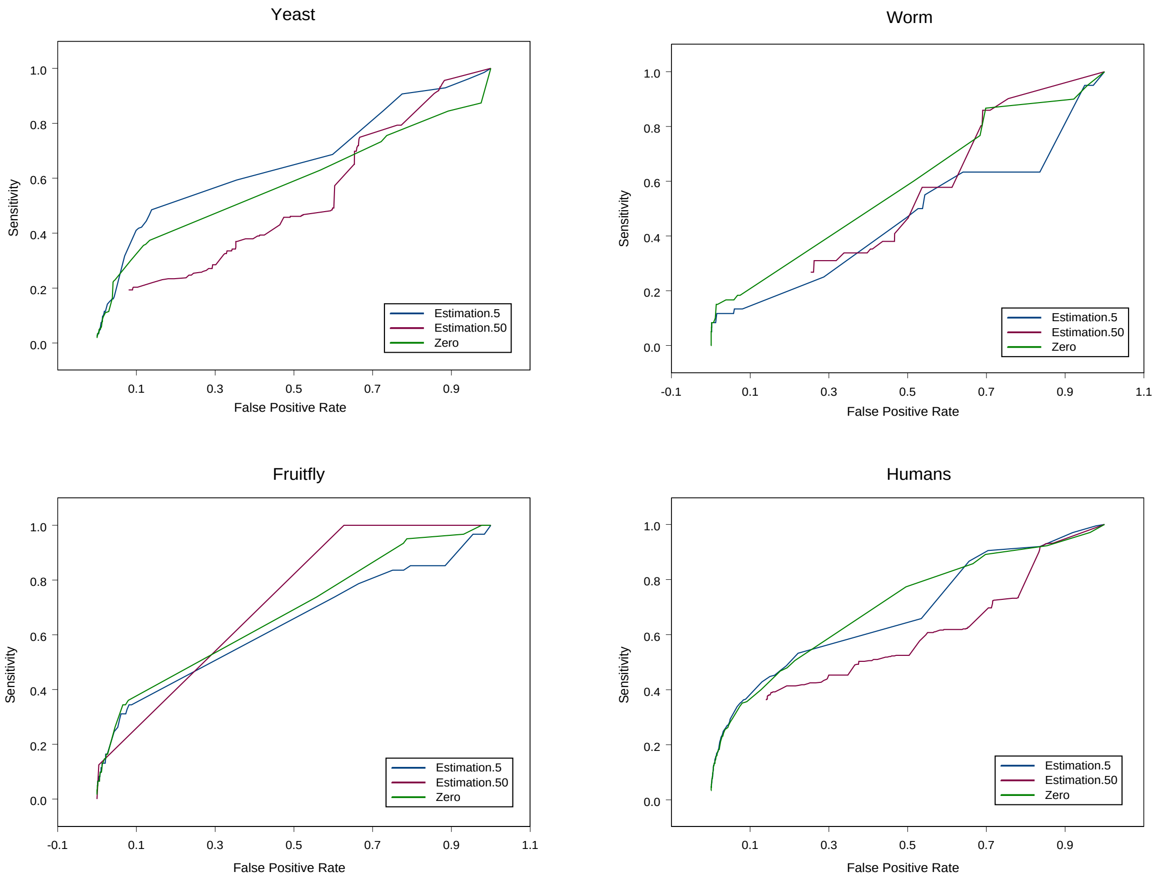

The relationship between false positive rate and sensitivity compared to the iPfam based on the "Expectation" score function with different fp and fn values: "Estimation.5" uses fp and fn estimated when t=5; "Estimation.50" uses fp and fn estimated when t=50; "Zero" uses fp=fn=0.

References

[1] Deng, M., Sun, F. and Chen, T. (2002) Inferring domain-domain interactions from protein-protein interactions. *Genome Res.*, **12**, 1540-1548.

[2] Lee, H.J., Deng, M., Sun, F.S., and Chen, T. (2005) Assessment of the Reliability of Protein-Protein Interactions Using Protein Localization and Gene Expression Data. *Bioinfo2005*, Busan.

[3] Hazbun, T. R. and Fields, S. (2001) Networking proteins in yeast. *Proc. Natl. Acad. Sci.*, **98**, 4277-4278.
