# Supplementary material for: An integrated approach to the prediction of domain-domain interactions
Source: BMC Bioinformatics. 2006 May 25;7:269. doi: 10.1186/1471-2105-7-269 (PMC1481624; doi:10.1186/1471-2105-7-269)
Supplement: Additional file 2 — The likelihood ratio of six data sources. The values for domain interactions inferred from six data sources are binned into discrete intervals and the likelihood ratio is calculated. [file 1471-2105-7-269-S2.pdf]

Table S1
The likelihood ratio of six data sources

Yeast	Gold-standard overlap				
E(#Dmn)	Obs	Nobs	P(yeast|Obs)	P(yeast|Nobs)	L
0--1	2354	47508	0.9121	0.9651	0.945
2--3	179	1562	0.0694	0.0317	2.186
4--5	17	73	0.0066	0.0015	4.441
6--7	14	41	0.0054	0.0008	6.512
8--9	4	14	0.0016	0.0003	5.449
10--11	3	3	0.0012	0.0001	19.072
12--inf	10	24	0.0039	0.0005	7.947
Sum	2581	49225			

Worm	Gold-standard overlap				
E(#Dmn)	Obs	Nobs	P(worm|Obs)	P(worm|Nobs)	L
0--1	2524	48472	0.9779	0.9847	0.993
2--3	38	549	0.0147	0.0112	1.320
4--5	12	152	0.0046	0.0031	1.506
6--7	0	36	0.0000	0.0007	0.000
8--9	2	4	0.0008	0.0001	9.536
10--11	0	8	0.0000	0.0002	0.000
12--inf	5	4	0.0019	0.0001	23.840
Sum	2581	49225			

Fruitfly	Gold-standard overlap				
E(#Dmn)	Obs	Nobs	P(fruitfly|Obs)	P(fruitfly|Nobs)	L
0--1	2522	48184	0.9771	0.9789	0.998
2--3	38	916	0.0147	0.0186	0.791
4--5	6	60	0.0023	0.0012	1.907
6--7	7	40	0.0027	0.0008	3.338
8--9	1	11	0.0004	0.0002	1.734
10--11	2	5	0.0008	0.0001	7.629
12--inf	5	9	0.0019	0.0002	10.596
Sum	2581	49225			

Human	Gold-standard overlap				
E(#Dmn)	Obs	Nobs	P(human|Obs)	P(human|Nobs)	L
0--1	2223	47560	0.8613	0.9662	0.891
2--3	208	1354	0.0806	0.0275	2.930
4--5	39	148	0.0151	0.0030	5.026
6--7	34	79	0.0132	0.0016	8.208
8--9	12	31	0.0046	0.0006	7.383
10--11	13	18	0.0050	0.0004	13.774
12--inf	52	35	0.0201	0.0007	28.336
Sum	2581	49225			

Coexist	Gold-standard overlap				
CE(Dmn)	Obs	Nobs	P(coexist|Obs)	P(coexist|Nobs)	LR
0	1440	46697	0.5579	0.9486	0.588
1--9	323	1668	0.1251	0.0339	3.693
10--19	92	339	0.0356	0.0069	5.176
20--29	70	111	0.0271	0.0023	12.027
30--39	42	78	0.0163	0.0016	10.270
40--49	48	49	0.0186	0.0010	18.683
50--99	176	133	0.0682	0.0027	25.238

100--149	116	62	0.0449	0.0013	35.683
150--inf	274	88	0.1062	0.0018	59.383
Sum	2581	49225			

SameGO	Gold-standard overlap				
SG(Dmn)	Obs	Nobs	P(SameGO|Obs)	P(SameGO|Nobs)	L
0	1279	34465	0.4955	0.7002	0.708
1--9	355	222	0.1375	0.0045	30.498
10--19	91	153	0.0353	0.0031	11.344
20--29	40	161	0.0155	0.0033	4.738
30--39	17	52	0.0066	0.0011	6.235
40--inf	799	14172	0.3096	0.2879	1.075
Sum	2581	49225			
