# Supplementary material for: An integrated approach to the prediction of domain-domain interactions
Source: BMC Bioinformatics. 2006 May 25;7:269. doi: 10.1186/1471-2105-7-269 (PMC1481624; doi:10.1186/1471-2105-7-269)
Supplement: Additional file 3 — Comparison with predicted conserved domain interactions and random interactions. Table S2 shows the significance of the number of predicted conserved domain interactions compared to the random interactions. [file 1471-2105-7-269-S3.pdf]

Table S2

The number of domain interactions from four species and the conserved domain interactions, along with that of random interactions by fixing the degree for each node in these domain interaction networks and shuffling edges. Also shown is the number of indirect interactions, which is obtained by connecting edges between domains having same interacting domains. The number of common direct interactions is more than three fold higher than that of random interactions. On the other hand, the number of common indirect interactions is two fold higher than that of random interactions, confirming the significance of common direct interactions. In both cases, the interaction networks contain 2,576 domains.

	Total	Common in more than one species	Ratio of common
Direct interactions	20,332	812	4.1%
Random direct interactions	20,803	244	1.2%
Indirect interactions	109,332	6,625	6.1%
Random indirect interactions	111,095	4,013	3.6%
