# Supplementary material for: An integrated approach to the prediction of domain-domain interactions
Source: BMC Bioinformatics. 2006 May 25;7:269. doi: 10.1186/1471-2105-7-269 (PMC1481624; doi:10.1186/1471-2105-7-269)
Supplement: Additional file 4 — List of conserved domain interactions predicted from protein interactions of at least three species. These conserved domain interaction have 31% of overlaps with domain interactions in iPfam. [file 1471-2105-7-269-S4.htm]

| Table S3 | | |  |  |  |  |  |  |
| List of conserved domain interactions predicted from protein interactions of at least three species. | | | | | | | | |
|  | | | | | | | | |
| PF00004 | PF00004 |  | | | | | | |
| PF00004 | PF00023 |  | | | | | | |
| PF00004 | PF00400 |  | | | | | | |
| PF00004 | PF00595 |  | | | | | | |
| PF00010 | PF00010 |  | | | | | | |
| PF00011 | PF00011 |  | | | | | | |
| PF00013 | PF00013 |  | | | | | | |
| PF00013 | PF00076 |  | | | | | | |
| PF00013 | PF00400 |  | | | | | | |
| PF00013 | PF01652 |  | | | | | | |
| PF00013 | PF04408 |  | | | | | | |
| PF00017 | PF00620 |  | | | | | | |
| PF00018 | PF00069 |  | | | | | | |
| PF00018 | PF00244 |  | | | | | | |
| PF00018 | PF02205 |  | | | | | | |
| PF00022 | PF00036 |  | | | | | | |
| PF00023 | PF00069 |  | | | | | | |
| PF00036 | PF00036 |  | | | | | | |
| PF00036 | PF00069 |  | | | | | | |
| PF00036 | PF00612 |  | | | | | | |
| PF00046 | PF00046 |  | | | | | | |
| PF00046 | PF00069 |  | | | | | | |
| PF00046 | PF00400 |  | | | | | | |
| PF00046 | PF00651 |  | | | | | | |
| PF00046 | PF02178 |  | | | | | | |
| PF00069 | PF00069 |  | | | | | | |
| PF00069 | PF00096 |  | | | | | | |
| PF00069 | PF00097 |  | | | | | | |
| PF00069 | PF00118 |  | | | | | | |
| PF00069 | PF00134 |  | | | | | | |
| PF00069 | PF00136 |  | | | | | | |
| PF00069 | PF00319 |  | | | | | | |
| PF00069 | PF00397 |  | | | | | | |
| PF00069 | PF00400 |  | | | | | | |
| PF00069 | PF00620 |  | | | | | | |
| PF00069 | PF00646 |  | | | | | | |
| PF00069 | PF00675 |  | | | | | | |
| PF00069 | PF00804 |  | | | | | | |
| PF00069 | PF00917 |  | | | | | | |
| PF00069 | PF01008 |  | | | | | | |
| PF00069 | PF01398 |  | | | | | | |
| PF00069 | PF02735 |  | | | | | | |
| PF00069 | PF02845 |  | | | | | | |
| PF00069 | PF03104 |  | | | | | | |
| PF00069 | PF03731 |  | | | | | | |
| PF00069 | PF03941 |  | | | | | | |
| PF00071 | PF00071 |  | | | | | | |
| PF00071 | PF00560 |  | | | | | | |
| PF00071 | PF00595 |  | | | | | | |
| PF00071 | PF00780 |  | | | | | | |
| PF00071 | PF01363 |  | | | | | | |
| PF00076 | PF00076 |  | | | | | | |
| PF00076 | PF00176 |  | | | | | | |
| PF00076 | PF00225 |  | | | | | | |
| PF00076 | PF00400 |  | | | | | | |
| PF00076 | PF04857 |  | | | | | | |
| PF00076 | PF07719 |  | | | | | | |
| PF00091 | PF00091 |  | | | | | | |
| PF00091 | PF03953 |  | | | | | | |
| PF00096 | PF00096 |  | | | | | | |
| PF00096 | PF00412 |  | | | | | | |
| PF00096 | PF00515 |  | | | | | | |
| PF00096 | PF00595 |  | | | | | | |
| PF00096 | PF00917 |  | | | | | | |
| PF00096 | PF03810 |  | | | | | | |
| PF00097 | PF00097 |  | | | | | | |
| PF00105 | PF03143 |  | | | | | | |
| PF00149 | PF04177 |  | | | | | | |
| PF00156 | PF00156 |  | | | | | | |
| PF00183 | PF00515 |  | | | | | | |
| PF00183 | PF03234 |  | | | | | | |
| PF00400 | PF02301 |  | | | | | | |
| PF00428 | PF00428 |  | | | | | | |
| PF00515 | PF00515 |  | | | | | | |
| PF00515 | PF04695 |  | | | | | | |
| PF00531 | PF00531 |  | | | | | | |
| PF00568 | PF00611 |  | | | | | | |
| PF00569 | PF00569 |  | | | | | | |
| PF00595 | PF02214 |  | | | | | | |
| PF00633 | PF00633 |  | | | | | | |
| PF00646 | PF01466 |  | | | | | | |
| PF00646 | PF03931 |  | | | | | | |
| PF00651 | PF00651 |  | | | | | | |
| PF00676 | PF02779 |  | | | | | | |
| PF00676 | PF02780 |  | | | | | | |
| PF00731 | PF00731 |  | | | | | | |
| PF00787 | PF00787 |  | | | | | | |
| PF00804 | PF00995 |  | | | | | | |
| PF00808 | PF02045 |  | | | | | | |
| PF01119 | PF01119 |  | | | | | | |
| PF01217 | PF01602 |  | | | | | | |
| PF01336 | PF01336 |  | | | | | | |
| PF01398 | PF01399 |  | | | | | | |
| PF01423 | PF01423 |  | | | | | | |
| PF01704 | PF01704 |  | | | | | | |
| PF01849 | PF01849 |  | | | | | | |
| PF01920 | PF02996 |  | | | | | | |
| PF02214 | PF02214 |  | | | | | | |
| PF02301 | PF05557 |  | | | | | | |
| PF02798 | PF02798 |  | | | | | | |
| PF02991 | PF03416 |  | | | | | | |
| PF03953 | PF03953 |  | | | | | | |
| PF04614 | PF04757 |  | | | | | | |
| PF07576 | PF07576 |  | | | | | | |
| PF07647 | PF07647 |  | | | | | | |
|  |  |  |  |  |  |  |  |  |
