# Supplementary material for: An integrated approach to the prediction of domain-domain interactions
Source: BMC Bioinformatics. 2006 May 25;7:269. doi: 10.1186/1471-2105-7-269 (PMC1481624; doi:10.1186/1471-2105-7-269)
Supplement: Additional file 5 — ROC curves of predicted domain interactions using yeast, worm, fruitfly and humans. Figure S2 shows the comparison of performances of score functions to predict domain interactions for four species. [file 1471-2105-7-269-S5.pdf]

The performance of each score function is evaluated by the ROC curve of sensitivity and false positive rate. For each score function and a threshold t, domain pairs with score at least t are predicted as interacting. The predicted domain interactions are compared with the domain interaction in iPfam to draw the ROC curve. Figure S2 shows the ROC curves based on the four different measures. The *expectation* approach gives the best prediction result for yeast and fruitfly. The accuracies of the *expectation* and *E-value* approaches are similar for humans. The *probability* approach gives the best result for worm.

# Figure S2

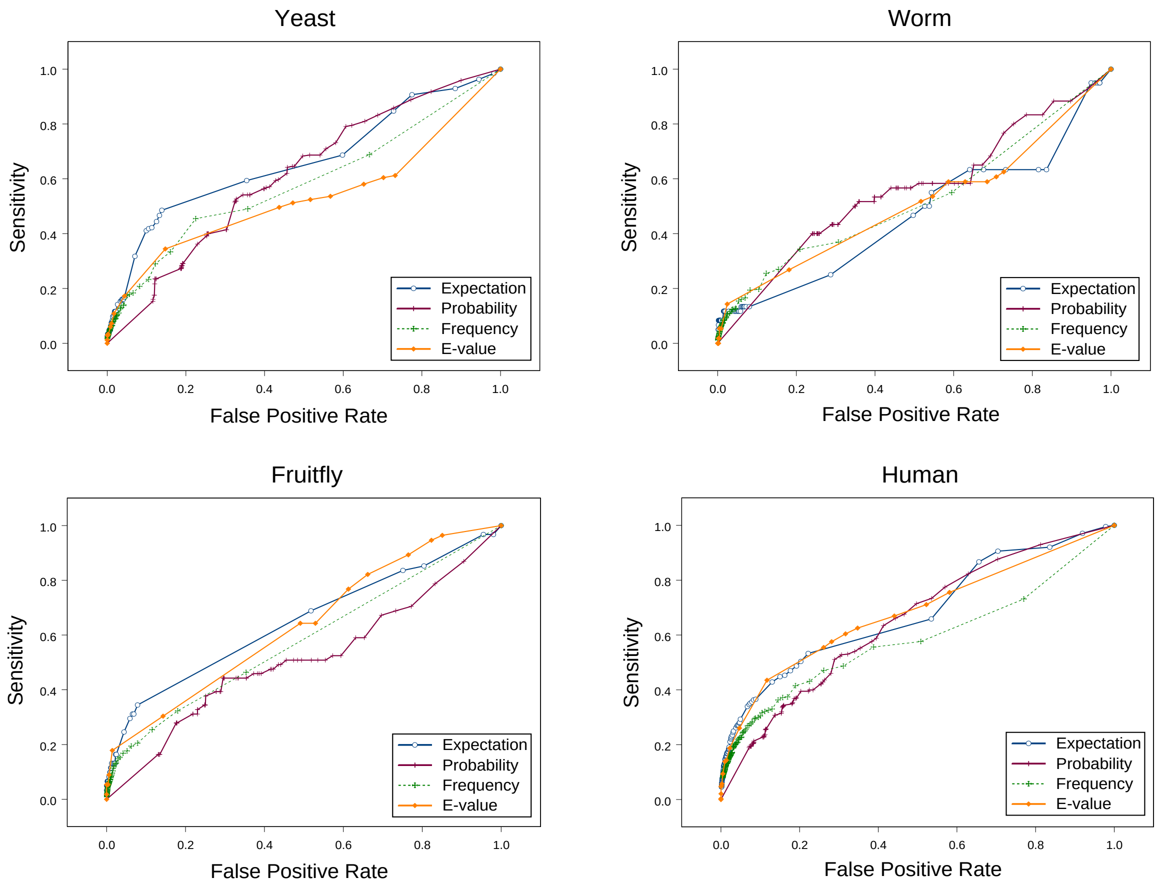


The relationship between false positive rate and sensitivity of predicted domain interactions compared to the iPfam for four species based on four score functions. "Expectation" ranks domain pairs according to the expected number of occurrences of domain pairs in protein interactions; "Probability" ranks domain pairs according to the estimated probability of interactions from the MLE method; "Frequency" ranks domain pairs according to the number of protein interactions having domain pair; "E-value" ranks domain pairs according to the E-value defined in Riley et al. [1].

# References

[1] Riley, R., Lee, C., Sabatti, C. and Eisenberg, D. (2005) Inferring protein domain interactions from databases of interacting proteins. *Genome Bio.*, **6(10)**, R89.
