# Supplementary material for: An integrated approach to the prediction of domain-domain interactions
Source: BMC Bioinformatics. 2006 May 25;7:269. doi: 10.1186/1471-2105-7-269 (PMC1481624; doi:10.1186/1471-2105-7-269)
Supplement: Additional file 8 — The likelihood ratio values of predicted domain interaction, the numbers of predicted domain interactions, and the overlap with domain interactions from H. pylori. We used 1,101 domain interactions in H. pylori involving 206 domains. Numbers in the first column indicate the likelihood ratio values for the domain interactions, and the second column is the number of interactions having the corresponding likelihood ratio values. "Fold" indicates the ratio of the fraction over expected value. (5.2%). [file 1471-2105-7-269-S8.pdf]

Table S6

The likelihood ratio values of predicted domain interaction, the numbers of predicted domain interactions, and the overlap with domain interactions from *H. pylori*. We used 1,101 domain interactions in *H. pylori* involving 206 domains. Numbers in the first column indicate the likelihood ratio values for the domain interactions, and the second column is the number of interactions having the corresponding likelihood ratio values. "Fold" indicates the ratio of the fraction over expected value (5.2%).

Likelihood ratio values	Interactions	Overlap with HPDDI	Fraction	Fold
Random domain pairs	21,321	1,101	5.2%	-
>0	1,640	119	7.3%	1.4
≥ 1	561	81	14.4%	2.8
≥ 4	346	73	21.1%	4.1
≥ 6	265	69	26.0%	5.0
≥ 11	242	65	26.9%	5.2
≥ 21	178	47	26.4%	5.1
≥ 51	117	34	29.1%	5.6
≥ 81	99	32	32.3%	6.3
