# Supplementary material for: An integrated approach to the prediction of domain-domain interactions
Source: BMC Bioinformatics. 2006 May 25;7:269. doi: 10.1186/1471-2105-7-269 (PMC1481624; doi:10.1186/1471-2105-7-269)
Supplement: Additional file 9 — A ROC curve of predicted domain interactions using H. pylori. Figure S3 shows the comparison of performances of score functions to predict domain interactions for H. pylori. [file 1471-2105-7-269-S9.pdf]

Figure S3

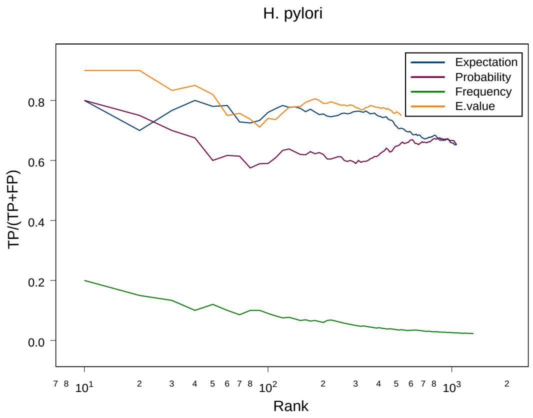

The relationship between rank and true positive rate (TP/(TP+FP)) compared to the domain interactions from *H. pylori* for predicted domain interactions from *H. pylori* based on four score functions. "Expectation" ranks domain pairs according to the expected number of occurrences of domain pairs in protein interactions; "Probability" ranks domain pairs according to the estimated probability of interactions from the MLE method; "Frequency" ranks domain pairs according to the number of protein interactions having domain pair; "E-value" ranks domain pairs according to the E-value defined in Riley et al. [1].

References

[1] Riley, R., Lee, C., Sabatti, C. and Eisenberg, D. (2005) Inferring protein domain interactions from databases of interacting proteins. *Genome Bio.*, **6(10)**, R89.
